# Impact of Grape Pomace Powder on the Phenolic Bioaccessibility and on In Vitro Starch Digestibility of Wheat Based Bread

**DOI:** 10.3390/foods10030507

**Published:** 2021-02-27

**Authors:** Gabriele Rocchetti, Corrado Rizzi, Mariasole Cervini, Giada Rainero, Federico Bianchi, Gianluca Giuberti, Luigi Lucini, Barbara Simonato

**Affiliations:** 1 Department for Sustainable Food Process, Università Cattolica del Sacro Cuore, Via Emilia Parmense 84, 29122 Piacenza, Italy; gabriele.rocchetti@unicatt.it (G.R.); luigi.lucini@unicatt.it (L.L.); 2 Department of Biotechnology, University of Verona, Strada Le Grazie 15, 37134 Verona, Italy; corrado.rizzi@univr.it (C.R.); mariasole.cervini@univr.it (M.C.); giada.rainero@univr.it (G.R.); federico.bianchi_02@univr.it (F.B.); barbara.simonato@univr.it (B.S.)

**Keywords:** bread fortification, grape pomace, foodomics, antioxidant activity, starch digestion, anthocyanins

## Abstract

Breads were prepared by substituting common wheat flour with 0 (GP0), 5 (GP5) and 10 (GP10) g/100 g (*w*/*w*) of grape pomace powder (GPP) and were analyzed for the phenolic profile bioaccessibility as well as the in vitro starch digestion during simulated digestion. The free and bound phenolic composition of native GPP and resulting breads were profiled using ultra-high-performance chromatography-quadrupole-time-of-flight (UHPLC-QTOF). The raw GPP was characterized by 190 polyphenols with the anthocyanins representing the most abundant class, accounting for 11.60 mg/g of cyanidin equivalents. Regarding the fortified bread, the greatest (*p* < 0.05) content in phenolic compounds was recorded for the GP10 sample (considering both bound and free fractions) being 127.76 mg/100 g dry matter (DM), followed by the GP5 (106.96 mg/100 g DM), and GP0 (63.76 mg/100 g DM). The use of GPP determined an increase of anthocyanins (considered the markers of the GPP inclusion), recording 20.98 mg/100 g DM in GP5 and 35.82 mg/100 g DM in GP10. The bioaccessibility of anthocyanins increased in both GP5 and GP10 breads when moving from the gastric to the small intestine in vitro digestion phase with an average value of 24%. Both the starch hydrolysis and the predicted glycemic index decreased with the progressive inclusion of GPP in bread. Present findings showed that GPP in bread could promote an antioxidant environment in the digestive tract and influence the in vitro starch digestion.

## 1. Introduction

Grape pomace (GP) represents the major residue from the winemaking process, accounting for up to the 30% of the original grape weight [1,2]. Nowadays, GP disposal represents a critical environmental issue. Nevertheless, recent attainments suggest of means of GP recovery with remarkable economic advantages [3]. GP contains considerable amounts of health-promoting components, including dietary fibers (up to 85% as a function of the grape variety) and polyphenolic compounds, which remain up to 70% after the winemaking process [4,5,6]. Hence, several food strategies have been proposed for GP reutilization, developing value-added products with possible health benefits [7].

In particular, GP has been successfully used in bread, biscuits, cereal bars and other cereal-based food products [6,8,9,10]. In all these studies, the GP inclusion improved the nutritional profile of the final products, thus contributing to formulating value-added foods with certain functional characteristics [2]. In this context, Tolve et al. [11] recently reported that the substitution of a part of common wheat flour in bread with GP powder (GPP; up to 10 g/100 g *w*/*w*) contributed to significantly increasing the total dietary fiber and the total polyphenol content, without impairing the acceptability scores with respect to white bread. Similar results have been reported by Hayta et al. [12].

However, most of the studies focused on the total phenolic content in the final products following GPP inclusion at different levels [6,8,9,10,11,12,13]. This may be of concern, since the possible health-promoting properties related to GPP polyphenols, and the effectiveness in food fortification, is not only related to their content in the final products, but also to the phenolic compounds’ bioaccessibility (and potential bioavailability) [5,13]. There is currently no information on changes in the phenolic profiles after in vitro digestion of GPP-enriched foods (i.e., bioaccessibility). Moreover, certain phenolic compounds characterizing GPP could have a role in modifying starch digestibly, by acting as potential starch enzyme inhibitors [10]. However, there is still limited information regarding the role of GPP inclusion in cereal-based foods on the digestibility of starch, at least in vitro.

Therefore, this research aimed to expand the knowledge concerning the use of GPP in bread formulation, by exploring the influence of increasing levels of GPP in wheat-based bread by focusing on (i) the phenolic profile and related bioaccessibility and (ii) the in vitro starch digestibility. To this end, breads were prepared with increasing substitution levels of GPP in the recipe (0, 5 and 10 g/100 g *w*/*w*). The GPP inclusion levels in the bread recipe were selected considering that the level up to 10 g/100 g did not impact the overall acceptability by consumers [11].

## 2. Materials and Methods 

### 2.1. Ingredients and Bread Preparation 

Grape pomace (*Vitis vinifera* cv. Corvina; Cantina Ripa della Volta, Verona, Italy) was treated as detailed by Cisneros-Yupanqui et al. [14]. Soft white wheat flour (WWF) was provided by Macinazione Lendinara SpA (Arcole, Italy). Experimental breads were formulated by replacing WWF with 0 (i.e., GP0), 5 (i.e., GP5) and 10 (i.e., GP10) g/100 g of GGP. In brief, 320 g of composite flours, 210 mL of tap water, 15 g of sugar, 3.5 g of dried brewer’s yeast and 3 g of salt were combined in a commercial breadmaking machine with operative conditions as follows: 39 min of dough fermentation at 28 °C, baking for 65 min at 170 °C. Three different doughs were made on the same day for each recipe. After baking and cooling, breads were cooled (i.e., room temperature) and kept in airtight food storage plastic bags. The chemical composition of major ingredients and the resulting breads has been reported elsewhere [11].

### 2.2. Extraction of Free and Bound Polyphenols from GPP and Experimental Breads

Free and bound phenolic extraction was conducted according to previously optimized conditions [15]. Three replicates (1.0 g) for each experimental bread and native GPP were extracted using an Ultra-Turrax (Ika T25, Staufen, Germany at 6000× *g* for 3 min) in a methanol/water solution (80:20 *v*/*v*; 10 mL) acidified with 0.1% formic acid. The extracts were centrifuged (10,000× *g*; 10 min; 4 °C), filtered (0.22 μm) and stored at −18 °C. Bound phenolics were extracted from the residual solids. Following an alkaline hydrolysis (3 mL of 2 M sodium hydroxide; 1 h; room temperature), the pH was adjusted to three (3 M citric acid) and the extraction was done in 8 mL of ethyl acetate. After 15 min at 6000× *g* centrifugation, 4 mL of the supernatant was dried under a nitrogen flow at 55 °C and the residue was dissolved in 1 mL of 0.1% formic acid in 80% methanol, vortexed and centrifuged (10,000× *g* for 10 min). The resulting solutions were filtered (0.22 μm) and an aliquot was transferred to amber vials.

### 2.3. In Vitro Static Digestion for the Evaluation of the Phenolic Modifications

The harmonized INFOGEST in vitro static digestion protocol was employed [16]. Briefly, for the oral phase, 100 g of each bread were moistened with 150 mL of a salivary fluid (SF; pH = 7.0; 37 °C) and chopped in a commercial meat grinder equipped with a 4 mm-hole plate. Twenty-five g of the resulting oral bolus was transferred in a 50 mL blue cap glass bottle, 5 mL of the SF containing α-amylase (A1031; Sigma-Aldrich; Milan, Italy; 150 U/mL; pH = 7.0; 37 °C) was added and then bottles containing-samples were incubated at 37 °C for 2 min. Then, 20 mL of a gastric fluid at pH 3.0 plus pepsin (P7012; Sigma-Aldrich; 2.000 U/mL) was added. This gastric phase lasted for 120 min at 37 °C. For the small intestine phase, 40 mL of an intestinal fluid at pH = 7.0 plus pancreatin (P7545; Sigma-Aldrich; Milan, Italy; 100 U/mL) and bile salts (B8631; Sigma-Aldrich; Milan, Italy; 10 mM) were added to the chyme and samples were further incubated for 120 min at 37 °C. In addition, HCl (1 M) and NaOH (1 M) were used for pH regulation. The composition of each simulated fluid was detailed by Minekus et al. [16]. Liquid aliquots were removed after the gastric and the small intestinal (i.e., pancreatic) phases and stored (i.e., −20 °C). Analyses were run in triplicate.

### 2.4. Phenolic Profiling by Ultra-High-Performance Liquid Chromatography-Quadrupole-Time-of-Flight (UHPLC-QTOF) Mass Spectrometry

The fate of polyphenols after each considered in vitro digestion phase (i.e., gastric and small intestine) was evaluated by ultra-high-performance liquid chromatography-quadrupole-time-of-flight (UHPLC-ESI/QTOF) mass spectrometry, as previously reported [15]. Collected liquid aliquots were centrifuged (7000× *g*; 10 min) and filtered (0.22 μm). The mobile phase was a mixture of acetonitrile and water (VWR, Milan, Italy) acidified with 0.1% formic acid (*v*/*v*). The chromatographic process was done on the Agilent Zorbax Eclipse-plus C18 column (100 mm × 2.1 mm, 1.8 μm) whilst a linear gradient from 6% acetonitrile to 94% acetonitrile, produced via a binary pump, was done in 34 min, considering flow rate of 0.22 mL/min. The mass spectrometer worked in the positive (ESI+) scan mode in the range 100–1200 *m*/*z*, injecting three replicates of 6 μL. The injection sequence was randomized, and quality control samples (QCs) were injected and acquired using a data-dependent auto-MS/MS mode using 12 precursors per cycle (1 Hz, 80–1200 *m/z*, positive polarity, active exclusion after two spectra). Collision energies of 10, 20 and 40 eV for collision-induced decomposition were used. The source conditions have been reported elsewhere [15,17].

The Agilent Profinder B.06 software was used to elaborate the raw mass features (i.e., entities consisting in a combination of raw abundances and isotopic mass spectra). Features were aligned, and the monoisotopic accurate mass was combined with the isotopic profile for the compounds’ annotation, thus reaching a level 2 of confidence in annotation (i.e., putatively annotated compounds). The annotation process was done with the database Phenol-Explorer 3.6, with a mass accuracy tolerance set to 5 ppm. Phenolic compounds passing the frequency of the detection thresholds (100% of replications within at least one condition) were classified and then quantitative information was produced using calibration curves (in the range 0.05–500 mg/L) from solutions of single standard phenolic compounds (Extrasynthese; Genay; France). Selected representative compounds were: cyanidin (anthocyanins), quercetin (flavanols and other flavonoids), luteolin (flavones), tyrosol (other lower molecular weight phenolics) and ferulic acid (phenolic acids). Results were expressed as mg phenolic equivalents/g DM. The software MS-DIAL (version 4.38) was then used to annotate compounds in QCs [18], using the MS/MS libraries provided by the same software (such as Mass Bank of North America). 

The polyphenols’ bioaccessibility was calculated according to the following equation:

*Bioaccessibility* = (PCA/PCB) × 100

where PCA is the total phenolic subclass content in the sample (mg/g DM) after each considered in vitro digestion phase, and PCB is the total phenolic subclass (free plus bound polyphenols) content in the same samples before the in vitro digestion [15].

### 2.5. In Vitro Antioxidant Capacity by ABTS and FRAP Assays

The in vitro antioxidant capacity was evaluated on the collected in vitro digested liquid aliquots by means of FRAP and ABTS assays, using the analytical conditions as specified by Tolve et al. [10]. For the ABTS assay, the absorbance was measured at 734 nm whereas for the FRAP assay at 593 nm. Results were expressed as micromolar of Trolox equivalent (TE)/100 g DM. 

### 2.6. In Vitro Starch Digestion and Calculations

One hundred mg of each bread was incubated with 4 mL of maleic buffer (pH 6.0), containing 4 μL of amyloglucosidase (300 U/mL) and 40 mg of pancreatic α-amylase (3000 U/mg) (Megazyme, Wicklow, Ireland) at 37 °C. After different incubation times (0, 20, 60, 120 and 180 min), 4 mL of pure ethanol was added to stop the reaction. After centrifugation (2500× *g*; 10 min), the amount of glucose was measured at 510 nm using a D-glucose assay kit (Megazyme, Wicklow, Ireland). The hydrolysis index (HI) was calculated as the percentage between the area under the hydrolyses curve (0–180 min) of the GPP-containing sample and the corresponding area of GP0. From the HI, a predicted glycemic index was determined with the formula of Granfeldt et al. [19] (pGI = 8.198 + 0.862 × HI).

### 2.7. Statistical Analyses 

Analyses were performed in triplicate. Data evaluation was made by analysis of variance (ANOVA) with a post hoc Tukey test (*p* < 0.05). XLSTAT Premium Version (2019.4.2, Addinsoft SARL, Paris, France) was used as statistical software. Data are reported as mean ± standard deviation.

The UHPLC-QTOF data were pre-processed with the software Agilent Mass Profiler Professional B.12.06 (Agilent Technologies, Santa Clara, CA, USA). Compounds were aligned, filtered by abundance (peak area > 2000 counts), normalized (75th percentile), and baselined against the median. Data on the phenolics annotated and their relative abundances were exported (SIMCA 13 software; Umetrics, Malmo, Sweden) to produce an OPLS-DA model (supervised orthogonal projection to latent structures discriminant analysis) for the evaluation of the changes of the phenolic profile. For cross validation of the OPLS-DA model, the Hotelling’s T2 together with CV-ANOVA (*p* < 0.01) and permutation testing were done. Model parameters as R^2^Y and Q^2^Y were recorded. Variables importance in projection (VIP) method was used to indicate the phenolic compounds with the highest discrimination ability (VIP score >1) through the in vitro digestion. 

## 3. Results and Discussion

### 3.1. Free and Bound Phenolic Profiles of GPP and GPP-Substituted Breads

The untargeted UHPLC-QTOF mass spectrometry was used to profile both free and bound phenolic composition of native GPP and the resulting GPP-breads. A comprehensive dataset reporting the identified compounds (i.e., according to a level 2 of confidence in annotation) is reported in Table S1. Overall, in raw GPP were identified 190 polyphenols, namely 39 anthocyanins, 10 flavan-3-ols, 31 flavones and derivatives, 36 flavanols, 28 low-molecular-weight phenolics (tyrosol derivatives) and 44 phenolic acids (mainly hydroxycinnamics). Those compounds belonging to different subclasses and presenting the highest abundance values were mainly malvidin 3-*O*-glucoside (in both free and bound fraction), followed by chrysoeriol 7-*O*-glucoside (characterizing the bound fraction), and different glycosidic forms of isorhamnetin. Regarding the semiquantitative contents (Table S1), GPP was characterized by 24.36 mg/g DM of total phenolic equivalents. Also, anthocyanins represented the most abundant class, accounting for 11.60 mg/g of cyanidin equivalents. In addition, the bound phenolic fraction represented only the 9% of the total phenolic content (i.e., 2.15 mg/g DM). According to literature, GP can be considered a great source of phenolic compounds with different bioactive properties [14]. According to Peixoto et al. [20] grape pomace is often undervalued but constitutes a potential source of polyphenols for possible food, pharmaceutical and cosmetic application. Also, the same authors reported a tentative identification of 28 phenolic compounds in the hydro-alcoholic extracts prepared from grape pomace mixture, seeds and skins, including flavan-3-ols (catechin derivatives), seven anthocyanins (such as delphinidin, malvidin, peonidin and petunidin derivatives), flavanols (mainly quercetin derivatives) and phenolic acids (both hydroxybenzoics and hydroxycinnamics). Therefore, our findings are matching with the corresponding scientific literature. In fact, according to previous works [20,21], the presence of these compounds (mainly anthocyanins in the grape pomace skins) is widely documented. 

**Figure 1:**
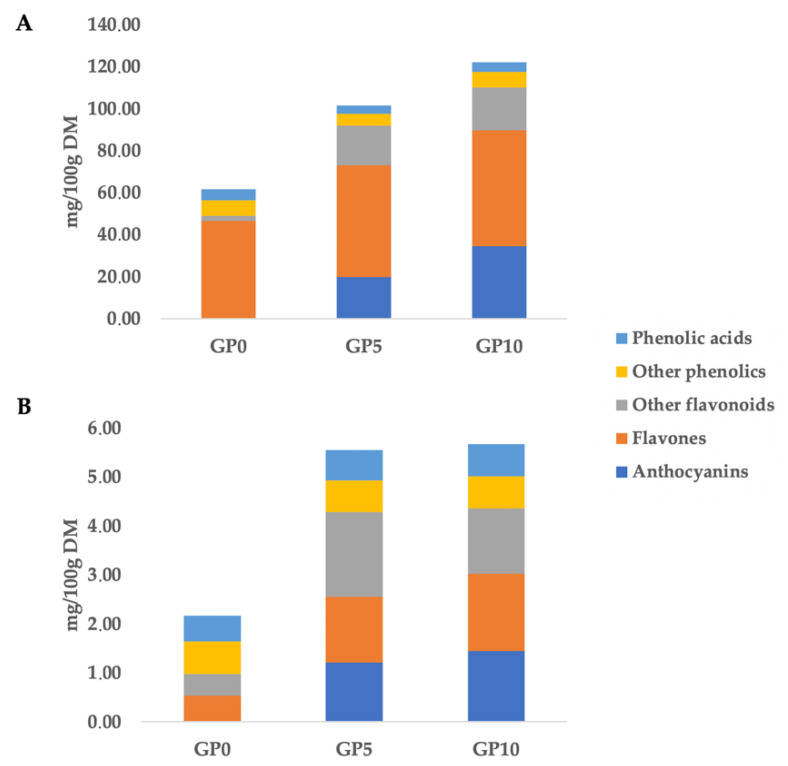
Cumulative phenolic composition (as mg phenolic equivalents/100 g dry matter) of control bread (GP0) and bread fortified with different inclusion level of grape pomace powder (5 g/100 g *w*/*w*: GP5; and 10 g/100 g *w*/*w*: GP10). Free (**A**) and bound (**B**) phenolic fractions.

Following the inclusion of different levels of GPP into WWF-based breads, the UHPLC-QTOF profiling allowed annotating 183 compounds (Table S1), with a great abundance of flavonoids (mainly flavone derivatives) and phenolic acids (mainly hydroxycinnamics). The cumulative phenolic composition of each experimental bread (i.e., GP0, GP5 and GP10) is reported in Figure 1. 

The greatest (*p* < 0.05) cumulative phenolic content (when considering both bound and free fractions) was found in GP10, being 127.76 mg/100 g DM, followed by the GP5 (106.96 mg/100 g DM) and GP0 (63.76 mg/100 g DM). These findings reflected the increasing substitution level of GPP to WWF in bread formulation. In particular, the phenolic profile most influenced by the GPP inclusion was that of the anthocyanins. The inclusion of GPP determined a great increase of anthocyanins, recording 20.98 mg/100 g DM in GP5 sample and 35.82 mg/100 g DM in GP10 (Figure 1). The bound phenolic profile was also influenced by the GPP inclusion level. In this regard, GP5 and GP10 experimental breads were characterized by a similar bound phenolic composition, recording an average of 5.61 mg/100 g DM and being about 2-fold greater than that of GP0. 

Overall, anthocyanins and other flavonoids (such as flavanols and flavan-3-ols) could be considered the markers of the GPP inclusion (Figure 1). Also, the untargeted phenolic profile revealed an abundance of flavones and derivatives in the WWF used as the major component in current bread formulation. In this regard, as can be observed in Figure 1, GP0 showed a high content of flavone equivalents, being 47.14 mg/100 g DM, with a great abundance of tetramethylscutellarein and cirsimaritin (Table S1), both belonging to methoxyflavones. These findings are in accordance with the phenolic composition typically reported for wheat grains [22], being composed mainly by flavones, phenolic acids and alkylphenols. Regarding targeted compounds, the GP0 sample was abundant in 5-nonadecenylresorcinol when considering both free and bound phenolic fractions, whilst malvidin 3-*O*-glucoside could be considered the most important marker of the inclusion of GPP in the formulation of experimental breads (Table S1). Finally, the annotation process based on QCs allowed us to identify an additional 114 compounds (Table S1), also confirming the structural identity of 11 phenolic compounds, namely cyanidin 3-*O*-glucoside, delphinidin 3-*O*-galactoside, peonidin 3-*O*-glucoside, dihydroquercetin, procyanidin dimer B1, epicatechin, genistein, isorhamnetin, quercetin 3-*O*-galactoside, coumarin and gallic acid. As indicated by Antonić et al. [2], the fortification of bread with GP represents an interesting topic in food fortification aiming to increase the total phenolic content of the final products.

### 3.2. Changes of Phenolic Bioaccessibility during the In Vitro Digestion

The GPP-breads were hydrolyzed through a standardized in vitro static digestion method, aiming to explain the changes in the phenolic profile. The UHPLC-QTOF untargeted profiling provided the identification of 109 polyphenols, with an abundance of flavonoids (59 compounds), phenolic acids (29 compounds) and lower-molecular-weight phenolics (21 compounds). Each annotated compound is reported in Table S1, together with its abundance values and composite mass spectrum. Tolve et al. [11], analyzing the same experimental breads, showed that the inclusion of higher levels of GPP caused an increase in the total dietary fiber content. The total dietary fiber content represents a key point when targeting the bioactive profile of a certain cereal-based food product. In particular, both chemical and physical characteristics of the food can affect the bioaccessibility, bioavailability and bioactivities of bioactive compounds, including polyphenols [23]. In particular, the phenolic compound bioavailability and bioaccessibility can be affected by molecular interactions between dietary fiber and the other components characterizing the food matrix (such as lipids and proteins) [24]. Therefore, in this work, the phenolic compounds exhibited different bioaccessibility trends, mainly imposed by the different percentage of dietary fiber in each bread sample, in line with previous indications for fortified wheat-based fresh pasta formulated with an increasing level of a high-fiber/polyphenols ingredient [15]. The bioaccessibility of anthocyanins was found to increase in both GP5 and GP10 experimental breads when moving from the gastric to the small intestine phase (Table 1). An average bioaccessibility value of 24% was recorded for this class of compounds.

The investigation of the anthocyanin stability in the gastrointestinal tract could be useful for exploring their final bioavailability and thus related potential health benefit. In particular, human in vitro digestion models demonstrated that anthocyanins are rather stable in the stomach, but easily degradable in the small intestine due to the high pH value [25]. The stability of anthocyanins in the small intestine is extremely important; indeed, as reviewed by Braga et al. [26], the gut microbiota has a pivotal role in the metabolism of anthocyanins reaching the colon because of its ability to produce lower molecular weight and more bioavailable compounds, such as caffeic acid, protocatechuic acid and 4-hydroxybenzoic acid [27]. 

Therefore, looking at our findings, we could speculate that the higher dietary fiber content of GPP-fortified breads determined a major carrier effect of anthocyanins in the large intestine, because of interactions between fiber and anthocyanins (mainly those glycosylated) [28], thus enhancing their potential bioaccessibility in the colon. Regarding the other subclasses of phenolic compounds under investigation, the inclusion of GPP determined a higher bioaccessibility of flavones after the small intestine phase for GP5 and GP10 when compared with the control (on average: 14.5% vs. 2.7%, respectively), whilst GP5 showed the highest bioaccessibility of the remaining flavonoids (i.e., other flavonoids), being 29.2% after the small intestine phase of the in vitro static digestion. It was also interesting to notice that the GP5 sample showed a high bioaccessibility value for lower-molecular-weight compounds, being 61.7% at the end of the small intestine phase (Table 1); in this regard, the most abundant compounds detected by UHPLC-QTOF (Table S1) were hydroxytyrosol, phlorin and 2,3-dihydroxy-1-guaiacylpropanone. Finally, similar bioaccessibility values were recorded for the phenolic acids when considering the small intestinal phase, ranging from 17% (GP0) up to 23.3% (GP10). Taken together, these findings showed that the relatively high percentage bioaccessibility values observed at the end of the small intestine phase for some phenolic classes (e.g., anthocyanins, flavones, and tyrosol equivalents) could promote an antioxidant environment in the digestive tract. 

No comprehensive work based on untargeted phenolic profiling coupled with in vitro digestion exists in literature for GPP-fortified breads. Regarding recent existing literature based on similar experimental designs, Kan et al. [29] evaluated the impact of berry polyphenols on starch digestion, testing both the co-digestion of a blueberry extract with bread and by fortifying bread with the same blueberry extract (considering 0%, 2.5%, and 5% inclusion levels). The authors reported a greater starch digestion inhibition effect for co-digesting berry extracts and bread than that obtained by digesting berry-fortified breads. Also, the interactions of polyphenols (mainly anthocyanins and procyanidins) with the food matrix reduced the polyphenols bioaccessibility, thus reducing the number of polyphenols potentially available for enzyme inhibition [29]. In another work, Lachowicz et al. [30] showed that the enrichment of rye breads with the 3% of a berry fruit powder determined a strong increase in the bioaccessibility of anthocyanins following an in vitro digestion process, whereas the least bioaccessible fraction was that of flavan-3-ols.

**Figure 2:**
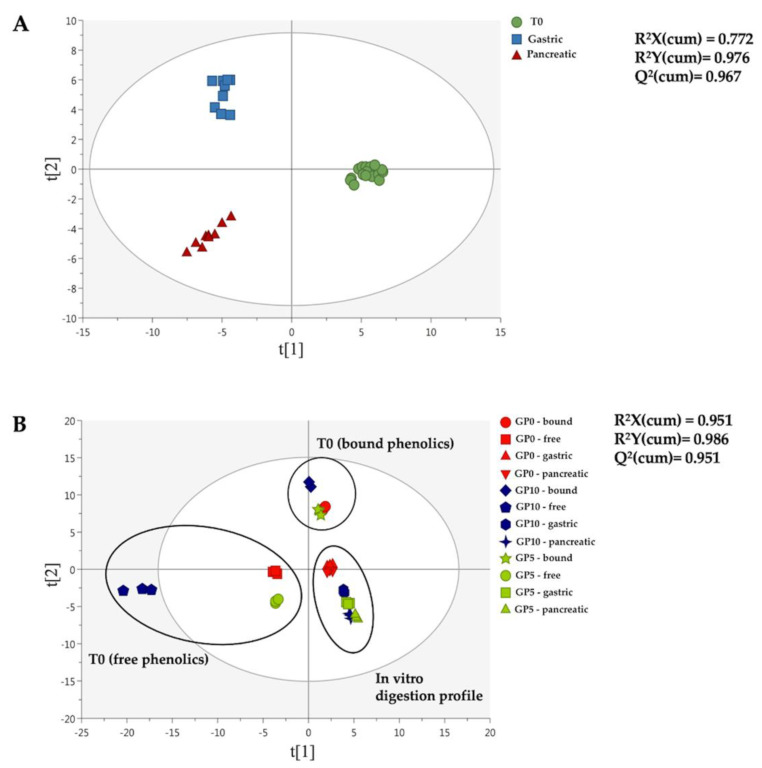
OPLS-DA score plots built considering both the digestion phase (**A**) and the matrix type (**B**) as class discrimination parameters. The dataset containing the annotated polyphenols characterizing the experimental breads (i.e., GP0, GP5 and GP10) before (T0) and after each phase of the in vitro static digestion (i.e., gastric and small intestine or pancreatic) was used to build the discriminant models. The cumulative goodness parameters of each model, namely R^2^X, R^2^Y and Q^2^, are provided.

Thereafter, multivariate statistics based on a supervised OPLS-DA approach was used to discriminate the different experimental breads during the in vitro digestion process, thus extrapolating the most discriminant compounds of the trends observed. To this aim, two different OPLS-DA score plots were produced and reported in Figure 2. 

Interestingly, both OPLS-DA models allowed the modification of phenolic profiles during the in vitro digestion process to be clearly observed, independently from the experimental bread (Figure 2A), thus revealing a strong impact of the gastrointestinal conditions in providing the detected modifications. On the other hand, the second OPLS-DA score plot (Figure 2B) showed a great difference between the free and the bound phenolic profiles of the experimental breads before the in vitro digestion (T0), with GP10 possessing the most characteristic phenolic profile. The second OPLS-DA score plot (Figure 2B) allowed to observe a completely different phenolic profile when comparing GP5 and GP10 to the control (GP0), and this was true at both in vitro gastric and small intestinal level. Interestingly, both the supervised models built were characterized by a high goodness of prediction (Q^2^ > 0.9) (Figure 2). Besides, the models presented a cross-validated *p*-value < 0.01, with no risk of overfitting (Table S1). Finally, the VIP selection method was used to rank those polyphenols most affected during the in vitro digestion process (i.e., considering the OPLS-DA model reported in Figure 2A). A comprehensive list containing these VIP discriminant markers is reported in Table S1, together with their corresponding VIP score and cross-validated standard error. Forty-two compounds were identified, comprising 15 flavonoids (mainly anthocyanins and flavones) and phenolic acids (mainly hydroxycinnamics). The highest VIP scores were recorded for *p*-coumaric acid (score = 1.74) and *p*-coumaroyl glycolic acid (score = 1.65). Also, the VIP approach revealed the presence of several anthocyanins, such as malvidin 3-*O*-glucoside (score = 1.05) that was previously highlighted as the marker of the GPP fortification.

### 3.3. Changes of Antioxidant Capacity during the In Vitro Static Digestion

In this work, the antioxidant capacity of the different bread samples before and after the simulated static digestion step was evaluated using two different in vitro assays, namely FRAP and ABTS (Table 2). Overall, the inclusion of GPP in bread determined an increase of both FRAP and ABTS values, likely due to the antioxidant activity of the anthocyanins characterizing the native GPP (i.e., 11.60 mg/g DM; Table S1). In this regard, anthocyanins have an antioxidant potential twice that of other known antioxidants, such as catechin (flavon-3-ols) [31]. Besides, looking at the antioxidant capacity values observed after the in vitro gastric and small intestine phases, an expected decrease of both ABTS and FRAP values was reported (Table 2); however, comparable activities were detected in both GP5- and GP10-fortified breads, thus confirming the bioaccessibility trends revealed by untargeted metabolomics (Table 1). Also, the reduction trends observed in both ABTS and FRAP activity values when moving from the gastric to the small intestine phases could be explained by a different pH stability of the phenolic compounds under investigation, or by the action of digestive enzymes. For example, it is known that the low pH value in the stomach can contribute to the high stability of anthocyanins, which at this pH value (i.e., 1–5.2) occurs as stable flavylium cation [25]. Therefore, looking to present findings, it is possible to hypothesize that the in vitro activities detected could be the results of complex interactions occurring between phenolic compounds and other food matrix components, to their chemical characteristics (i.e., degree of glycosylation), and to a different stability during the simulated gastrointestinal digestion conditions.

### 3.4. In Vitro Starch Digestion of the GPP-Fortified Breads

The hydrolysis index (HI) can be used to calculate a predicted glycemic index (pGI) values of starch-based foods and to predict the in vivo glycemic response of a food of interest [31]. In general, WWF-based breads are characterized by high HI and pGI values [32]. As reported in Table 3, both the HI and pGI values showed a decrease (*p* < 0.05) with the progressive inclusion of GPP in bread. In particular, the HI and the corresponding pGI decreased from 100 to 86.45 and from 94.20 to 82.72, respectively for GP0 and GP10. Similar results have been reported in cooked wheat pasta samples formulated with increasing amount of GPP in the recipe (from 0 to 10 g/100 g *w*/*w*) [10]. This can be related to changes in the chemical composition, as well as to possible interactions among the different constituents within the food matrix, following the inclusion level of GPP in a WWF-based bread recipe [11]. In particular, some of the starch granules could be subjected to a limited starch gelatinization, due to the dietary fiber content from GPP, which may compete with starch for water absorption and/or limit the extent of gelatinization through starch encapsulation during hydrothermal processing [33]. Moreover, dietary fiber cell wall component can encapsulate starch granules, thus influencing the in vitro starch digestibility through a limited access to digestive enzymes [34]. The denser matrix of GP5 and GP10 breads as compared to GP0 by means of texture analysis [11] could have also contributed to decrease the in vitro starch digestion [33,34,35,36].

Lastly, the inherent phenolic profile of the GPP, mainly phenolic acids, tannins and flavonoids, could reduce the in vitro starch digestibility, by inhibiting starch hydrolyzing enzymes and/or through non-covalent interactions with starch on cooking, leading to the formation of starch-complexes with partial enzyme accessibility [37]. Accordingly, Hanhineva et al. [38] showed that different classes of polyphenols can inhibit the activities of α-amylase and α-glucosidase in vitro. Also, polyphenols may limit the speed and the degree of starch gelatinization by hydrogen bonding between phenol and amylose molecules [39]. Similar results were observed in samples of bread fortified with quinoa flour [40] and mango peel powder [41], as well as in wheat-based fresh pasta formulated with increasing levels of *Moringa oleifera* leaf powder [15]. 

## 4. Conclusions

The GPP, used for bread fortification (up a level of 10% *w*/*w*), was characterized by 39 anthocyanins, 10 flavan-3-ols, 31 flavones and derivatives, 36 flavonols, 28 low-molecular-weight phenolics (tyrosol derivatives), and 44 phenolic acids (mainly hydroxycinnamics). The greatest content in phenolic compounds was recorded for the G10 sample being 127.76 mg/100 g DM. The fortification with GPP determined a great increase of anthocyanins (recording 35.82 mg/100 g DM in GP10) whose bioaccessibility, after in vitro digestion, increased when moving from the gastric to the small intestine phase with an average value of 24%. Moreover, the increasing level of GPP in bread determined a decrease in the starch hydrolysis and in the predicted glycemic index. These results together with the data that we previously reported regarding the technological and sensorial aspects indicate that GPP can be used as valuable ingredient to produce value-added wheat bread.

## Figures and Tables

**Table 1:** Cumulative and average (n = 3) phenolic contents and bioaccessibility values (expressed as % of phenolic equivalents) for the different subclasses during the in vitro static digestion of samples formulated with different substitution levels of grape pomace powder (i.e., 0, 5 and 10 g/100 g for GP0, GP5 and GP10, respectively), considering the cumulative phenolic contents of samples prior to digestion and after the gastric and the small intestine digestion phases. The results are expressed as mg/100 g DM of material prior to digestion. Nd: not detected.

Phenolic Subclass	Bread Sample	Prior to Digestion (mg/100 g DM)	Gastric (mg/100 g DM)	Small Intestine (mg/100 g DM)
Anthocyanins	GP0	nd	nd	nd
	GP5	20.98	3.18 (15.1%)	5.88 (28%)
	GP10	35.82	3.57 (10%)	7.25 (20.2%)
Flavones	GP0	47.14	1.77 (3.8%)	1.25 (2.7%)
	GP5	54.68	4.23 (7.7%)	9.33 (17.1%)
	GP10	56.80	4.57 (8%)	6.74 (11.9%)
Other flavonoids	GP0	2.85	nd	0.53 (18.7%)
	GP5	20.44	4.54 (22.2%)	5.97 (29.2%)
	GP10	21.86	4.28 (19.6%)	5.13 (23.5%)
Other phenolics	GP0	8.05	1.75 (21.7%)	2.14 (26.6%)
	GP5	6.30	1.46 (23.2%)	3.89 (61.7%)
	GP10	7.94	1.60 (20.2%)	3.08 (38.8%)
Phenolic acids	GP0	5.71	0.57 (10.1%)	0.97 (17%)
	GP5	4.57	0.39 (8.5%)	0.94 (20.6%)
	GP10	5.35	0.71 (13.2%)	1.25 (23.3%)

**Table 2:** In vitro antioxidant activity (as FRAP and ABTS) of control bread (GP0) and bread fortified with different percentages of grape pomace powder (GP5: 5 g/100 g GPP; and GP10: 10 g/100 g GPP) before and after the gastric and small intestine phases of in vitro simulated digestion.

Activity	Bread Sample	Prior to Digestion (µM TE/100 g DM)	Gastric (µM TE/100 g DM)	Small Intestine (µM TE/100 g DM)
FRAP	GP0	199.72 ± 9.69 ^a^	40.12 ± 3.18 ^a^	53.71 ± 5.37 ^a^
	GP5	795.26 ± 63.11 ^b^	169.27 ± 17.93 ^b^	100.22 ± 9.59 ^b^
	GP10	1577.39 ± 87.20 ^c^	231.44 ± 12.43 ^c^	135.65 ± 12.05 ^c^
ABTS	GP0	240.00 ± 7.90 ^a^	150.94 ± 14.08 ^a^	54.47 ± 3.08 ^a^
	GP5	999.50 ± 24.78 ^b^	369.17 ± 11.40 ^b^	223.76 ± 13.13 ^b^
	GP10	1540.83 ± 47.45 ^c^	426.46 ± 10.34 ^c^	233.00 ± 14.44 ^c^

Values with different superscripts within the same column are significantly different for *p* < 0.05.

**Table 3:** Hydrolysis index (HI) and predicted glycemic index (pGI) of control bread (GP0) and bread fortified with different percentages of grape pomace powder (GP5: 5 g/100 g GPP; and GP10:10 g/100 g GPP). Values are expressed as mean ± standard deviation.

Bread Sample	HI	pGI
GP0	100.00 ± 0.00 ^a^	94.20 ± 0.00 ^a^
GP5	93.57 ± 0.62 ^b^	88.86 ± 0.54 ^b^
GP10	86.45 ± 2.30 ^c^	82.72 ± 1.98 ^c^

The values with different superscripts within the same column are significantly different for *p* < 0.05.

## Data Availability

Not applicable.

## Supplementary Materials

The following are available online at https://www.mdpi.com/2304-8158/10/3/507/s1, Table S1: (a) Phenolic profile by UHPLC-QTOF of the GPP; (b) free and bound phenolic contents as mg/g of the GPP; (c) free and bound phenolic contents as mg/g of the different bread samples; (d) phenolic profile by UHPLC-QTOF of the different bread samples during in vitro digestion; (e) validation parameters and VIP discriminant compounds of the OPLS-DA models considering the digestion process; (f) Compounds structurally confirmed by UHPLC-QTOF-MSMS experiments, using the annotations provided by the QC samples; (g) Representative total ion current (TIC) chromatograms and extracted ion current plots for some GP10 phenolics when considering gastric and small intestine phases of in vitro gastrointestinal digestion.
